# Non-Motor Symptoms in Patients Suffering from Motor Neuron Diseases

**DOI:** 10.3389/fneur.2016.00117

**Published:** 2016-07-25

**Authors:** René Günther, Nicole Richter, Anna Sauerbier, Kallol Ray Chaudhuri, Pablo Martinez-Martin, Alexander Storch, Andreas Hermann

**Affiliations:** ^1^Department of Neurology, Division for Neurodegenerative Diseases, Technische Universität Dresden, Dresden, Germany; ^2^Department of Basic and Clinical Neuroscience, The Maurice Wohl Clinical Neuroscience Institute, King’s College London, London, UK; ^3^National Center of Epidemiology and CIBERNED, Carlos III Institute of Health, Madrid, Spain; ^4^Department of Neurology, University of Rostock, Rostock, Germany; ^5^German Center for Neurodegenerative Diseases (DZNE) Dresden, Dresden, Germany

**Keywords:** amyotrophic lateral sclerosis, motor neuron disease, NMSQuest, non-motor symptoms, multisystem disorder

## Abstract

**Background:**

The recently postulated “disease spreading hypothesis” has gained much attention, especially for Parkinson’s disease (PD). The various non-motor symptoms (NMS) in neurodegenerative diseases would be much better explained by this hypothesis than by the degeneration of disease-specific cell populations. Motor neuron disease (MND) is primarily known as a group of diseases with a selective loss of motor function. However, recent evidence suggests disease spreading into non-motor brain regions also in MND. The aim of this study was to comprehensively detect NMS in patients suffering from MND.

**Methods:**

We used a self-rating questionnaire including 30 different items of gastrointestinal, autonomic, neuropsychiatric, and sleep complaints [NMS questionnaire (NMSQuest)], which is an established tool in PD patients. 90 MND patients were included and compared to 96 controls.

**Results:**

In total, MND patients reported significantly higher NMS scores (median: 7 points) in comparison to controls (median: 4 points). Dribbling, impaired taste/smelling, impaired swallowing, weight loss, loss of interest, sad/blues, falling, and insomnia were significantly more prevalent in MND patients compared to controls. Interestingly, excessive sweating was more reported in the MND group. Correlation analysis revealed an increase of total NMS score with disease progression.

**Conclusion:**

NMS in MND patients seemed to increase with disease progression, which would fit with the recently postulated “disease spreading hypothesis.” The total NMS score in the MND group significantly exceeded the score for the control group, but only 8 of the 30 single complaints of the NMSQuest were significantly more often reported by MND patients. Dribbling, impaired swallowing, weight loss, and falling could primarily be connected to motor neuron degeneration and declared as motor symptoms in MND.

## Introduction

Motor neuron diseases (MND), such as amyotrophic lateral sclerosis, are neurodegenerative diseases with a progressive degeneration of motor neurons and their axons. Consecutive loss of motor function like paralysis of extremities and impairment of the respiratory apparatus are the cardinal symptoms. For many neurodegenerative diseases, it is well known that the pathology is not limited to the initially affected cell populations. Instead, disease spreading and involvement of other non-motor regions in the brain seem to occur (1). Corresponding non-motor symptoms (NMS) like gastrointestinal-, autonomic-, neuropsychiatric-, and sleep disorders are well known in these diseases. Treatments of such NMS are fundamental elements in a modern and comprehensive health care for patients suffering from neurodegenerative diseases (2, 3).

Frequently reported comorbidity with frontotemporal dementia (FTD), autonomic dysfunctions like delayed gastric emptying and colonic transit times, small-fiber neuropathy, pain as well as abnormalities in extra-motor areas in functional brain imaging studies make it justified to classify also MND as multisystem disorders (4–10). Moreover, recent neuropathology studies showed disease spreading also in MND and FTD, suggesting the involvement of non-motor regions in the pathophysiology of these disorders (11–13).

The aim of this study was to investigate the appearance of NMS in MND by comparing their prevalence to age- and sex-matched healthy individuals using the NMS questionnaire (NMSQuest). Although the NMSQuest was designed and validated for Parkinson’s disease (PD) (2), this questionnaire with its 30 items represents a suitable screening tool for NMS in MND.

## Patients and Methods

### Patients

Patients with definite, probable, or possible amyotrophic lateral sclerosis, according to the revised El Escorial criteria (14, 15), as well as patients with primary lateral sclerosis, were recruited from 2012 to 2015 at the Department of Neurology of the University Hospital Dresden. Patients suffering from genetic variants of spinal muscular atrophy, spinal bulbar muscular atrophy, and FTD overlap syndromes were not included. Additionally, age- and sex-matched controls were recruited from several centers (Europe, USA, and Japan) who participated in the NMSQuest study (3). The study was approved by the institutional review board at the Technische Universität Dresden (EK 393122012, EK 49022016). The initial ethical approval was obtained by the research ethics committee at the University Hospital Lewisham and subsequently all involved centers obtained site-specific ethical approvals.

### Assessments

The NMSQuest is a 30-item self-completed questionnaire featuring responses as “yes” and “no” to each item and was originally designed and validated for PD patients (2, 3, 16). These 30 items can be grouped into 9 domains (digestive, urinary, memory, perceptions, mood, sex, cardiovascular, sleep, and miscellaneous), as previously described (2, 3). “Total NMS score” was defined by the calculated sum of all positive (“yes”) answers of the 30 items. We additionally recorded age, gender, current symptoms with a special focus on bulbar symptoms, MND subtype, vital capacity, body weight, disease duration, and the revised ALS-Functional-Rating-Scale (ALSFRS-R). A sub-cohort was retested after a period of 6 ± 3 months.

### Statistical Analysis

As the samples were not normally distributed, statistical comparisons of data between groups were made using the non-parametric Mann–Whitney *U*-test (MWU) for total NMS score, NMS domains, and age. The chi-squared test (χ^2^) was carried out for a comparison of gender distribution and for a comparison of the proportion of “yes” and “no” responses between patients and controls for each single item. Spearman rank correlation coefficients were used to examine correlations between total NMS score and demographic as well as clinical data in the MND group with a correlation coefficient of rho < 0.3 considered as a weak, rho = 0.3–0.59 a moderate, and rho ≥ 0.6 a strong correlation. Data were analyzed using the software programs SPSS 21.0 (SPSS Inc., Chicago, IL, USA) and Statistica 12.0 [StatSoft (Europe) GmbH, Hamburg, Germany]. If not mentioned otherwise, all data are displayed as means ± SD. Significance level was set at *p* < 0.05. Correction for multiple testing was not applied as the study was merely exploratory, and a previous hypothesis was not proposed.

## Results

NMSQuest data from 90 MND patients and 96 controls were analyzed and compared. Demographic and clinical characteristics of the NMS study populations are shown in Table 1. The two groups did not differ significantly in age (MWU *p* = 0.87) or gender (χ^2^ *p* = 0.78).

**Table 1:** **Demographic and clinical characteristics of the study populations**.

	Control group	MND group
Total number	96	90
Ratio of females in %	52.1	50.0
Age in years	65.3 ± 10.5	65.1 ± 10.5
Disease duration in years	–	3 ± 2.3
ALS-FRS-R (range)	–	30 (8–46)
Subtypes (*n*)	–	Spinal onset (53)
		Bulbar onset (26)
		Primary lateral sclerosis (11)
Bulbar symptoms in %	–	66.7

**Figure 1:**
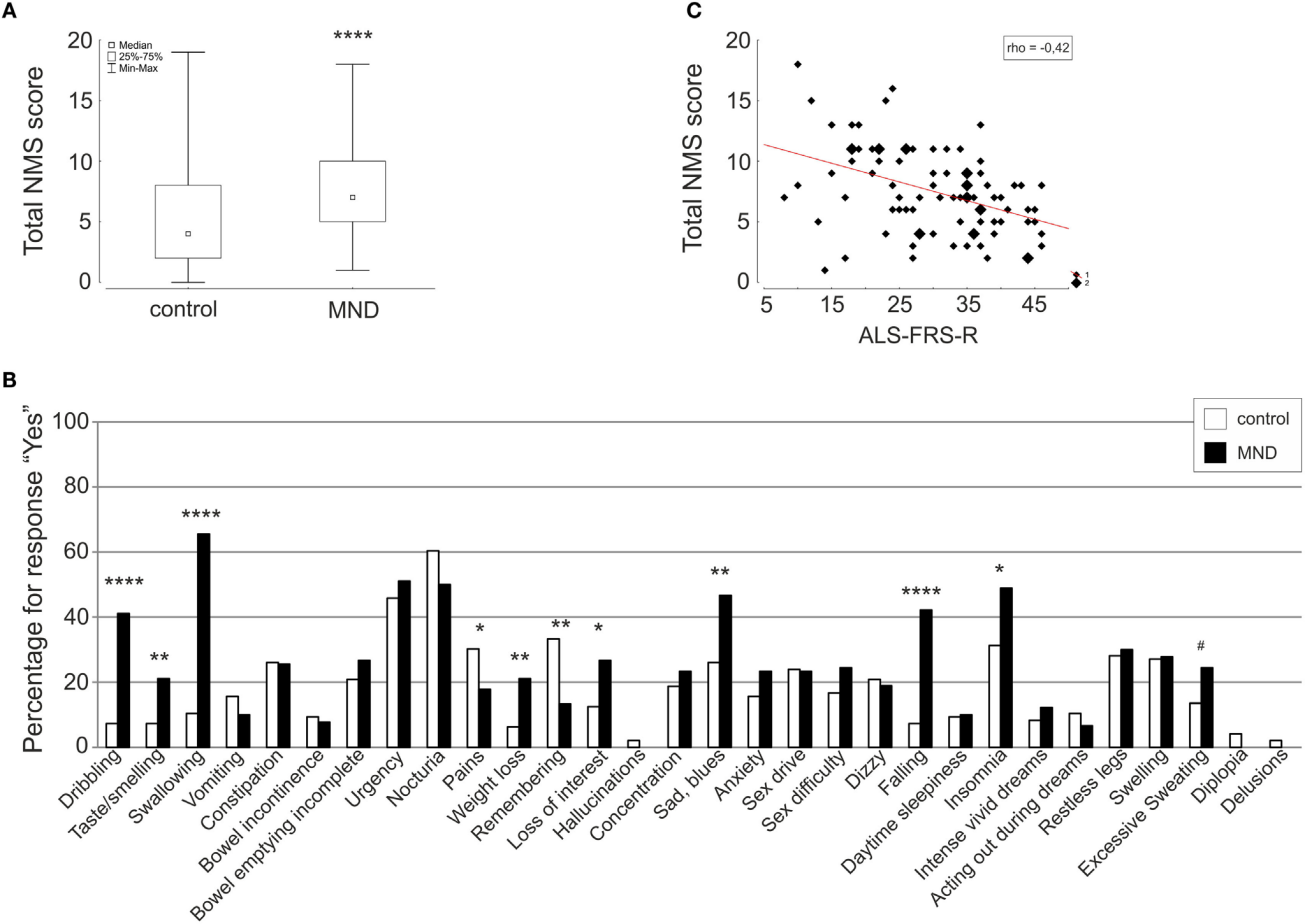
**Comparison of total NMS score (A) and single items (B) of the NMSQuest between controls (white) and MND patients (black) depicted as box plot and histograms, respectively**. Correlation of total NMS score and ALSFRS-R is shown as scatterplot **(C)**. Spearman rank correlation coefficient (rho), *p < 0.05, **p < 0.01, ***p < 0.001, ****p < 0.0001, ^#^*p* < 0.06 (trend).

Motor neuron disease patients reported significantly more NMS in total compared to healthy individuals (Figure 1A). The sum of NMS ranged from 1 to 18 with median 7 for MND patients and from 0 to 19 in the control group with median 4 (MWU *p* < 0.0001).

Comparisons on the NMS domain level showed significantly higher scores for MND patients in the domains “digestive” (MND: 1.98 ± 1.54; control: 0.97 ± 1.26; MWU *p* < 0.00001), “mood” (MND: 0.70 ± 0.79; control: 0.42 ± 0.71; MWU *p* < 0.01), and “cardiovascular” (MND: 0.61 ± 0.67; control: 0.28 ± 0.56; MWU *p* < 0.001).

On the single-item level, MND patients reported significantly more dribbling, impaired taste/smelling, impaired swallowing, weight loss, loss of interest, sad/blues, falling, and insomnia. Excessive sweating was 11% more often reported in the MND group compared to the control group. The items pain and remembering were significantly less reported by MND patients. Detailed statistics are presented in Table 2 and Figure 1B.

**Table 2:** **Comparison of responses between controls and MND patients**.

	Distribution of response “yes” in percentages		Chi-squared-test
	Control group	MND group	For yes–no
Dribbling	7.3	41.1	0.00000
Taste/smelling	7.3	21.1	0.0066
Swallowing	10.4	65.6	0.00000
Vomiting	15.6	10.0	0.25
Constipation	26.0	25.6	0.94
Bowel incontinence	9.4	7.8	0.69
Bowel emptying	20.8	26.7	0.35
Urgency	45.8	51.1	0.47
Nocturia	60.4	50.0	0.15
Pains	30.2	17.8	0.048
Weight loss	6.3	21.1	0.003
Remembering	33.3	13.3	0.001
Loss of interest	12.5	26.7	0.015
Hallucinations	2.1	0.0	n.c.
Concentration	18.8	23.3	0.44
Sad, blues	26.0	46.7	0.003
Anxiety	15.6	23.3	0.18
Sex drive	24.0	23.3	0.92
Sex difficulty	16.7	24.4	0.19
Dizzy	20.8	18.9	0.74
Falling	7.3	42.2	0.00000
Daytime sleepiness	9.4	10.0	0.86
Insomnia	31.3	48.9	0.014
Intense vivid dreams	8.3	12.2	0.38
Acting out during dreams	10.4	6.7	0.36
Restless legs	28.1	30.0	0.77
Swelling	27.1	27.8	0.92
Excessive sweating	13.5	24.4	0.057
Diplopia	4.2	0.0	n.c.
Delusions	2.1	0.0	n.c.

*n.c, non-calculable*.

Since some of the above mentioned NMS (swallowing, dribbling, and taste/smelling) could be due to bulbar paresis, we separately analyzed these items in MND patients without bulbar symptoms (MNDwbs). By doing so, there were no longer any significant difference to controls regarding dribbling (MNDwbs: 6.7%; control: 7.3%; χ^2^ *p* = 0.9) and impaired taste/smelling (MNDwbs: 16.7%, control: 7.3%; χ^2^ *p* = 0.12). However, significantly more patients in the “MNDwbs” group complained about impaired swallowing as compared to the control group (MNDwbs: 26.7%; control: 10.4%; χ^2^ *p* = 0.02).

Correlation analysis of total NMS score to clinical and demographic data revealed a moderate correlation with ALS-FRS-R (rho = −0.42, *p* = 0.00005) (Figure 1C) and a weak correlation with age (rho = 0.24, *p* = 0.03) and vital capacity (rho = −0.27, *p* = 0.02).

Follow-up data after 6 ± 3 months of 43 MND patients showed an increase of total NMS score (0.95 ± 2.64). The increase of total NMS score correlated with the decrease of ALS-FRS-R (rho = 0.34, *p* = 0.03) and the decrease of vital capacity (rho = 0.39, *p* = 0.03).

## Discussion and Conclusion

Non-motor symptoms are very common in PD and other neurodegenerative disorders (2, 3, 17). Frontotemporal impairment and memory loss are commonly known as “non-motor” involvements in MND patients. Besides that, multiple other extra-motor symptoms in MND have been reported in the last years (18), for example, autonomic dysfunctions (4, 19–21), sensory symptoms due to distal small-fiber neuropathy (5), and pain (6–8). However, this is the first pilot study for a comprehensive detection of NMS in MND patients.

Dribbling, impaired taste/smelling, impaired swallowing, weight loss, loss of interest, sad/blues, falling, excessive sweating, and insomnia were found to be prominent complaints in MND patients compared to controls. Dribbling, impaired swallowing, weight loss, and falling could be primarily discussed as motor symptoms. In line with that, dribbling was reported with equal frequency by MNDwbs compared to controls in the current study. Investigations in salivary gland function revealed saliva flow rate alterations, but these abnormalities were mild in contrast to the relevant problem of dribbling in MND patients suggesting that this symptom primarily is a consequence of bulbar motor impairment (20). Nevertheless, swallowing was still reported significantly more often by MND patients without clinically detectable bulbar symptoms. Presumably, subjective impairment of the swallowing act exists in some patients before bulbar symptoms are observed in clinical examination.

Taste/smelling complaints were significantly more reported, however only in patients suffering from bulbar symptoms. Hyposmia and hyperechogenic substantia nigra are well-known predictive markers for PD (22). Interestingly, hyperechogenicity of the substantia nigra was recently reported also in MND (23, 24). Thus, future studies regarding hyposmia in MND are of interest for interpreting possible similarities to PD. To our knowledge, large case–control studies with diagnostic tests for hyposmia in MND have not been previously reported.

Insomnia could be regarded as NMS but could partly be due to insufficient respiratory function during sleep and could therefore be discussed as a motor symptom. As confirmed by our results, affection of mood is a well-known problem in the treatment of MND patients (25–27). Moreover, we found excessive sweating increased in the patients as compared to the controls. This fits to recent data showing abnormal sympathetic activity with hyperhidrosis in early MND and a reduction in sweat production with disease progression (28). Pain was significantly less reported in our study. Detailed and specific pain questionnaires already revealed the relevant impact of pain in MND (6–8), but pain in these patients is mostly a result of immobility, muscle atrophy as well as increased muscle tone and could be due to motor neuron degeneration. The question about pain in the NMSQuest aimed at “unexplained pain,” which could be confusing and lead to a false negative answer. Furthermore, the diversity of pain may not be answered in a single question. Thus, pain in the domain of NMS needs future investigation. The item impaired remembering was significantly less common in the MND group in our study, whereas an increased frequency of cognitive impairment has previously been shown in MND patients (29).

Previous investigations have suggested that quality of life in MND is not strongly related to the level of physical impairment and that MND patients displace their focus away from decreasing health status (30, 31). These coping mechanisms possibly reduce the self-perception of NMS in MND patients. We therefore cannot exclude that some of the items, which do not differ between the patient and control group in our study, can be relevant after detailed interview. Future studies with objective measurement methods are therefore necessary.

To summarize, the total amount of NMS among the MND patients exceeded the amount of such symptoms in the control group. The single-item analysis revealed relevant complaints in MND patients, and the total NMS score seemed to increase with disease progression. However, comparing total NMS of MND patients with the already published data of PD patients, the impact of NMS in MND (median 7) turned out to be less pronounced than in PD (median 9) (2). On the single-item level, the PD group reported more complaints in 16 different items compared to controls, while for MND, we only detected significant differences for 8 items. Additionally, the most prominent items dribbling, impaired swallowing, weight loss, and falling are primarily caused by degeneration of the motor neurons. Nevertheless, NMS seem to increase with disease progression, which would fit with recently published neuropathological data, suggesting spreading of disease with widespread neuropathology at advanced stages (11–13). Therefore, it would be of great interest to perform another study on the frequency of NMS in a group of patients representing advanced MND disease stages. Finally, we strongly believe that assessment and treatment of NMS should be included in a modern and comprehensive health care of MND patients.

## Author Contributions

RG: conception and design, collection and assembly of data, data analysis and interpretation, and manuscript drafting. NR, AS, KC, and PM-M: collection and/or assembly of data and critical revision of manuscript. AS: conception and design, collection and/or assembly of data, data analysis and interpretation, and critical revision of manuscript. AH: conception and design, principal investigator, collection and assembly of data, data analysis and interpretation, and manuscript drafting.

## Conflict of Interest Statement

The authors declare that the research was conducted in the absence of any commercial or financial relationships that could be construed as a potential conflict of interest.

## Abbreviations

χ^2^, chi-squared test; ALSFRS-R, revised ALS-Functional-Rating-Scale; FTD, frontotemporal dementia; MND, motoneuron disease; MNDwbs, MND patients without bulbar symptoms; MWU, Mann–Whitney *U*-test; NMS, non-motor symptoms; PD, Parkinson’s disease.

## References

[1] Brundin P Melki R Kopito R. Prion-like transmission of protein aggregates in neurodegenerative diseases. Nat Rev Mol Cell Biol (2010) 11:301–7. 10.1038/nrm2873 20308987 PMC2892479

[2] Chaudhuri KR Martinez-Martin P Schapira AH Stocchi F Sethi K Odin P International multicenter pilot study of the first comprehensive self-completed nonmotor symptoms questionnaire for Parkinson’s disease: the NMSQuest study. Mov Disord (2006) 21:916–23. 10.1002/mds.20844 16547944

[3] Chaudhuri KR Martinez-Martin P Brown RG Sethi K Stocchi F Odin P The metric properties of a novel non-motor symptoms scale for Parkinson’s disease: results from an international pilot study. Mov Disord (2007) 22:1901–11. 10.1002/mds.21596 17674410

[4] Toepfer M Folwaczny C Klauser A Riepl RL Muller-Felber W Pongratz D. Gastrointestinal dysfunction in amyotrophic lateral sclerosis. Amyotroph Lateral Scler Other Motor Neuron Disord (1999) 1:15–9. 10.1080/146608299300079484 12365061

[5] Truini A Biasiotta A Onesti E Di Stefano G Ceccanti M La Cesa S Small-fibre neuropathy related to bulbar and spinal-onset in patients with ALS. J Neurol (2015) 262:1014–8. 10.1007/s00415-015-7672-0 25683764

[6] Wallace VC Ellis CM Burman R Knights C Shaw CE Al-Chalabi A. The evaluation of pain in amyotrophic lateral sclerosis: a case controlled observational study. Amyotroph Lateral Scler Frontotemporal Degener (2014) 15:520–7. 10.3109/21678421.2014.951944 25204842

[7] Chio A Canosa A Gallo S Moglia C Ilardi A Cammarosano S Pain in amyotrophic lateral sclerosis: a population-based controlled study. Eur J Neurol (2012) 19:551–5. 10.1111/j.1468-1331.2011.03540.x 21972798

[8] Wicks P Reassessing received wisdom in ALS – pain is common when studied systematically. Eur J Neurol (2012) 19:531–2. 10.1111/j.1468-1331.2011.03541.x 21972839

[9] van der Graaff MM Sage CA Caan MW Akkerman EM Lavini C Majoie CB Upper and extra-motoneuron involvement in early motoneuron disease: a diffusion tensor imaging study. Brain (2011) 134:1211–28. 10.1093/brain/awr016 21362631

[10] van der Graaff MM de Jong JM Baas F de Visser M. Upper motor neuron and extra-motor neuron involvement in amyotrophic lateral sclerosis: a clinical and brain imaging review. Neuromuscul Disord (2009) 19:53–8. 10.1016/j.nmd.2008.10.002 19070491

[11] Braak H Brettschneider J Ludolph AC Lee VM Trojanowski JQ Del Tredici K Amyotrophic lateral sclerosis-a model of corticofugal axonal spread. Nat Rev Neurol (2013) 9:708–14. 10.1038/nrneurol.2013.221 24217521 PMC3943211

[12] Brettschneider J Del Tredici K Toledo JB Robinson JL Irwin DJ Grossman M Stages of pTDP-43 pathology in amyotrophic lateral sclerosis. Ann Neurol (2013) 74:20–38. 10.1002/ana.23937 23686809 PMC3785076

[13] Kassubek J Muller HP Del Tredici K Brettschneider J Pinkhardt EH Lule D Diffusion tensor imaging analysis of sequential spreading of disease in amyotrophic lateral sclerosis confirms patterns of TDP-43 pathology. Brain (2014) 137:1733–40. 10.1093/brain/awu090 24736303

[14] Brooks BR El Escorial World Federation of Neurology criteria for the diagnosis of amyotrophic lateral sclerosis. Subcommittee on Motor Neuron Diseases/Amyotrophic Lateral Sclerosis of the World Federation of Neurology Research Group on Neuromuscular Diseases and the El Escorial “Clinical limits of amyotrophic lateral sclerosis” workshop contributors. J Neurol Sci (1994) 124(Suppl):96–107. 7807156 10.1016/0022-510x(94)90191-0

[15] Brooks BR Miller RG Swash M Munsat TL El Escorial revisited: revised criteria for the diagnosis of amyotrophic lateral sclerosis. Amyotroph Lateral Scler Other Motor Neuron Disord (2000) 1:293–9. 10.1080/146608200300079536 11464847

[16] Storch A Odin P Trender-Gerhard I Fuchs G Reifschneider G Ray Chaudhuri K Non-motor symptoms questionnaire and scale for Parkinson’s disease. Cross-cultural adaptation into the German language. Nervenarzt (2010) 81:980–5. 10.1007/s00115-010-3010-z 20414634

[17] Klingelhoefer L Samuel M Chaudhuri KR Ashkan K. An update of the impact of deep brain stimulation on non motor symptoms in Parkinson’s disease. J Parkinsons Dis (2014) 4:289–300. 10.3233/JPD-130273 24613865

[18] Swinnen B Robberecht W. The phenotypic variability of amyotrophic lateral sclerosis. Nat Rev Neurol (2014) 10:661–70. 10.1038/nrneurol.2014.184 25311585

[19] Nubling GS Mie E Bauer RM Hensler M Lorenzl S Hapfelmeier A Increased prevalence of bladder and intestinal dysfunction in amyotrophic lateral sclerosis. Amyotroph Lateral Scler Frontotemporal Degener (2014) 15:174–9. 10.3109/21678421.2013.868001 24479577

[20] Baltadzhieva R Gurevich T Korczyn AD. Autonomic impairment in amyotrophic lateral sclerosis. Curr Opin Neurol (2005) 18:487–93. 10.1097/01.wco.0000183114.76056.0e 16155429

[21] Pavlovic S Stevic Z Milovanovic B Milicic B Rakocevic-Stojanovic V Lavrnic D Impairment of cardiac autonomic control in patients with amyotrophic lateral sclerosis. Amyotroph Lateral Scler (2010) 11:272–6. 10.3109/17482960903390855 20001491

[22] Lerche S Seppi K Behnke S Liepelt-Scarfone I Godau J Mahlknecht P Risk factors and prodromal markers and the development of Parkinson’s disease. J Neurol (2014) 261:180–7. 10.1007/s00415-013-7171-0 24190794

[23] Hermann A Reuner U Schaefer J Fathinia P Leimert T Kassubek J The diagnostic value of midbrain hyperechogenicity in ALS is limited for discriminating key ALS differential diagnoses. BMC Neurol (2015) 15:33. 10.1186/s12883-015-0280-x 25879789 PMC4379542

[24] Fathinia P Hermann A Reuner U Kassubek J Storch A Ludolph AC. Parkinson’s disease-like midbrain hyperechogenicity is frequent in amyotrophic lateral sclerosis. J Neurol (2013) 260:454–7. 10.1007/s00415-012-6654-8 22923257

[25] Atassi N Cook A Pineda CME Yerramilli-Rao P Pulley D Cudkowicz M. Depression in amyotrophic lateral sclerosis. Amyotroph Lateral Scler (2011) 12:109–12. 10.3109/17482968.2010.536839 21091399 PMC3155886

[26] Lulé D Häcker S Ludolph A Birbaumer N Kübler A. Depression and quality of life in patients with amyotrophic lateral sclerosis. Dtsch Arztebl Int (2008) 105:397–403. 10.3238/arztebl.2008.0397 19626161 PMC2696844

[27] Kurt A Nijboer F Matuz T Kübler A. Depression and anxiety in individuals with amyotrophic lateral sclerosis: epidemiology and management. CNS Drugs (2007) 21:279–91. 10.2165/00023210-200721040-00003 17381183

[28] Beck M Giess R Magnus T Puls I Reiners K Toyka KV Progressive sudomotor dysfunction in amyotrophic lateral sclerosis. J Neurol Neurosurg Psychiatry (2002) 73:68–70. 10.1136/jnnp.73.1.68 12082050 PMC1757317

[29] Murphy J Factor-Litvak P Goetz R Lomen-Hoerth C Nagy PL Hupf J Cognitive-behavioral screening reveals prevalent impairment in a large multicenter ALS cohort. Neurology (2016) 86:813–20. 10.1212/WNL.0000000000002305 26802094 PMC4793785

[30] Fegg MJ Kogler M Brandstatter M Jox R Anneser J Haarmann-Doetkotte S Meaning in life in patients with amyotrophic lateral sclerosis. Amyotroph Lateral Scler (2010) 11:469–74. 10.3109/17482961003692604 20235757

[31] Tramonti F Bongioanni P Di Bernardo C Davitti S Rossi B. Quality of life of patients with amyotrophic lateral sclerosis. Psychol Health Med (2012) 17:621–8. 10.1080/13548506.2011.651149 22313252

